# An ethnobotanical study of wild edible plants used by the Tibetan in the Rongjia River Valley, Tibet, China

**DOI:** 10.1186/s13002-023-00621-4

**Published:** 2023-10-27

**Authors:** Jin Wang, Xiaoyong Ding, Chang-An Guo, Xiong Zhang, Haowen Feng, Huizhao Yang, Yuhua Wang

**Affiliations:** 1 Department of Economic Plants and Biotechnology, Yunnan Key Laboratory for Wild Plant Resources, Kunming Institute of Botany, Chinese Academy of Sciences, 132 Lanhei Road, Heilongtan, Yunnan, 650201 Kunming China; 2 University of Chinese Academy of Sciences, Beijing, China; 3 National Centre for Borderland Ethnic Studies in Southwest China, Yunnan University, Kunming, 650091 China; 4 College of Life Sciences, Shaanxi Normal University, Xi’an, 710119 China

**Keywords:** Tibetan, Wild edible plants, Ethnobotany, Traditional knowledge, Everest region

## Abstract

**Background:**

Wild edible plants (WEPs) play a crucial role in communities with limited communication with the outside world, where unstable factors, such as poor food supply and insufficient access to timely nutritional supplementation, are common, as in the Himalayan region. To document the traditional knowledge of WEPs and explore their significance for communities with minimal global economic exchange, an ethnobotanical study was conducted in the town of Rongjia, which lies in a narrow valley near Mount Everest, Tibet, China.

**Methods:**

This ethnobotanical study was conducted in three villages in the Rongjia River Valley between August 2021 and June 2023. Semi-structured interviews and participatory observations were used to collect information on WEPs. The fieldwork was performed with the assistance of local guides. Voucher specimens were collected from each documented plant species for taxonomic identification. We used the use report (UR) and relative frequency of citations (RFC) to evaluate the comprehensive utilization value of WEPs.

**Results:**

We interviewed 161 informants who provided us with 2499 use reports. We collected 50 WEPs belonging to 28 families and 42 genera used by the Tibetan people in the Rongjia River Valley. WEPs are used in vegetables, fruits, seasonings, healthcare foods, substitute grains, and beverages. Wild vegetables were the most commonly used, followed by wild fruits. Leaves were the most commonly consumed part of the plant. The three most important WEPs ordered by RFC values were *Rosa sericea* var. *glandulosa* Osmaston (RFC = 0.76), *Zanthoxylum bungeanum* Maxim. (RFC = 0.75), and *Urtica hyperborea* Jacquem. ex Wedd. (RFC = 0.71). Other than that, we also document some of WEPs used in the past. *Arisaema erubescens* Schott, *Pinellia ternata* (Thunb.) Makino, and *Satyrium nepalense* var. *ciliatum* (Lindl.) Hook. f. used to serve as important substitute grains, are no longer in use, however, they remain vivid in the memories of older people.

**Conclusions:**

WEPs included wild vegetables, fruits, seasonings, healthcare food, and substitute grains for Tibetan people in the Rongjia River Valley. Some WEPs have become important cultural symbols for older people, which can help in understanding the relationship between plants and local people in the past. In addition, WEPs can increase the resilience of local people living in remote areas when facing sudden destabilizing events in future. This is the significance of WEPs for communities with minimal global economic exchange. Therefore, we suggest that future studies focus more on WEPs in communities with limited communication with the world to improve their resilience.

## Background

The Mount Everest Reserve, the world’s highest mountain nature reserve, is one of the world’s biodiversity hotspots [1, 2]. The core region of the Mount Everest Reserve is one of the most remote and underdeveloped areas in China and worldwide. Residents in this area are exposed to harsh ecological environments with high altitude, strong ultraviolet light, lack of oxygen, low temperature, and barren land for cultivation [2]. Faced with these conditions, native people are heavily dependent on natural resources for their livelihoods [3]. When the food supply for farming is insufficient, wild edible plants (WEPs), which are non-cultivated and non-domesticated plants, play an indispensable role in the daily diet of many remote and underdeveloped regions [4–9].

Extreme geographical and ecological conditions not only isolate plant populations [10] and result in culturally unique biodiversity [11–13], but also shape the traditional uses of WEPs [9, 14]. In recent years, researchers have conducted many ethnobotanical studies on the southern slopes of the Himalayas [4–8, 15, 16], including Mêdog County [17], Chenthang Township [5], Burang Township [7], Yadong River Valley [4], Gyirong River Valley [6, 8], and other areas [16, 18–20]. For example, a field survey was conducted on the Monpa people in Mêdog County, using the changes in the plant knowledge mastered by the Monpa people to explore the changes in the local social and economic areas [17]. In order to cope with the seasonal food shortage caused by traffic blockage and scarce arable land, the Chenthang Sherpa people use WEPs as seasonal food supplements [5]. Moreover, WEPs provide a survival guarantee for the local Tibetan and Daman people in the Gyirong River Valley [6, 8]. These studies suggest that in underdeveloped and remote areas, WEPs play an important role in maintaining a stable food supply and the nutritional value of a balanced diet.

The Rongjia River Valley, located in the core area of Mount Everest Reserve, is a rare Xanadu Valley on the Qinghai-Tibet Plateau [21]. It is one of the China-Nepal and South Asia channels from China to Nepal [22]. It has a high mountain and deep valley terrain with an elevation span of more than 5,000 m and features a distinctive and diverse ecology, resulting in a unique three-dimensional climate, abundant plant resources, and high levels of biodiversity [23]. Because of the complex terrain and high altitude, the cultivated land area in the Rongjia River Valley is quite limited, and its agricultural yield is low. In addition, the supply of external vegetables and fruits is unstable due to inconvenient roads and the accidental influence of natural disasters. As a result, over their long history, the Tibetan people, combining their environmental conditions, religious beliefs, and cultural customs, have formed a set of unique traditional food cultures with benign interactions with the natural ecological environment [24]. Even now, to enter the area, one has to climb 5,400 m of snowy mountains, and access to goods, materials, and information from the outside world is still limited by environmental conditions. It is necessary to understand how local Tibetans recognize and use plants in their surrounding environments to improve their resilience. Consequently, the purpose of this research is to investigate, collect, and document WEPs and their traditional knowledge in the Rongjia River Valley. The aim was to understand why the Tibetan people in the Rongjia River Valley choose these plants and explore the significance of WEPs for communities with minimal global economic exchange.

## Methods

### Study area

**Fig. 1:**
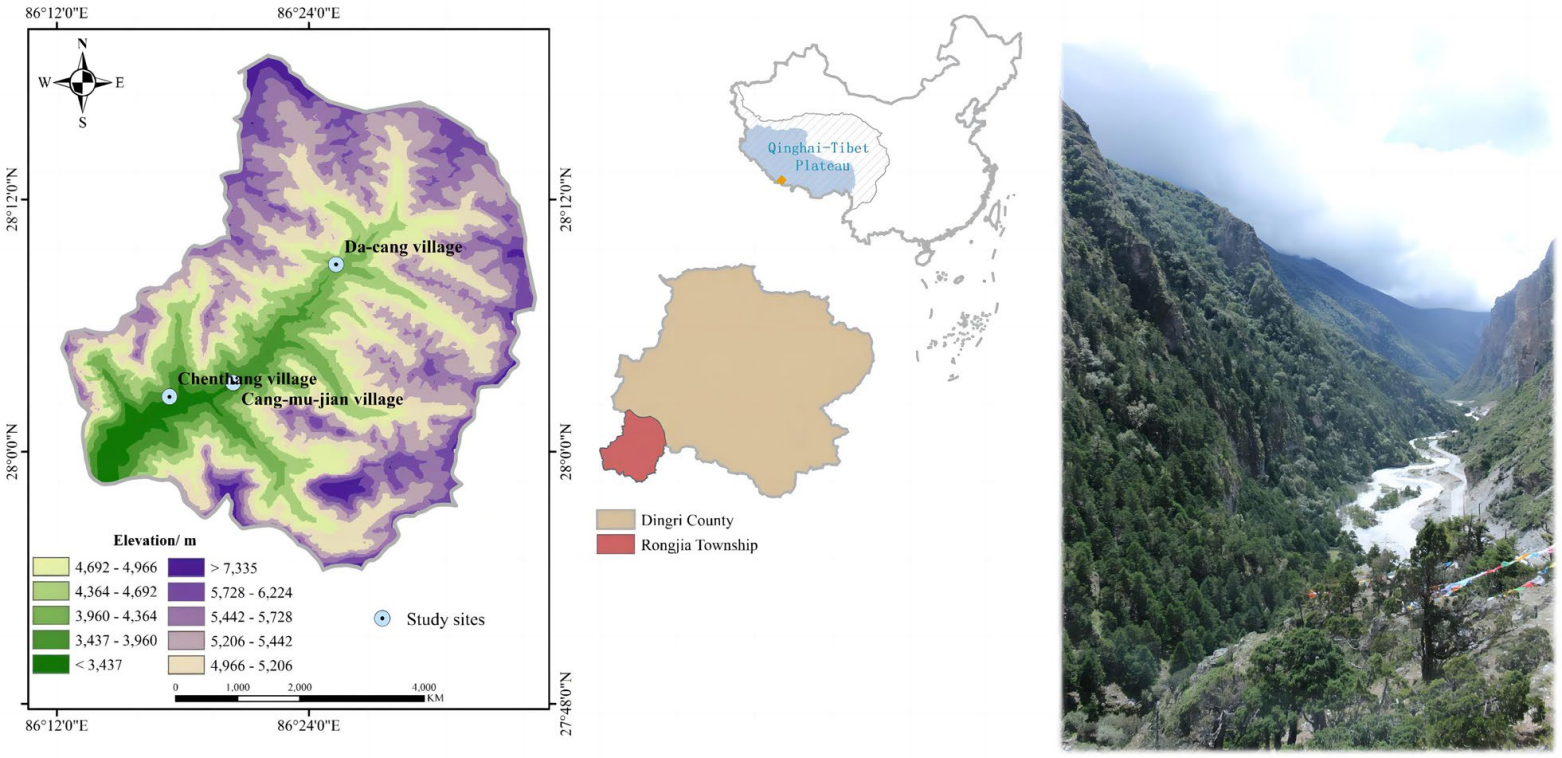
The location of survey sites

The Rongjia Township is located in Mount Qomolangma National Nature Reserve in the southeast of the Qinghai-Tibet Plateau on the China–Nepal border, southwest of Dingri County, Xigaze City, and the Tibet Autonomous Region (Fig. 1). The total area was nearly 980 square kilometers, the forest coverage rate was 60%, and the total forest area was 12,252.87 hectares. The cultivated land area was 48.6 hectares, and the grain-sown area was only 24.93 hectares.

The terrain of the Rongjia River Valley is a high mountain and deep valley landform with an elevation from 2500 to 7400 m [25]. The entire area of the township is significantly affected by warm and humid airflow from the Indian Ocean [25]. It belongs to a valley-cutting climate area, which is a typical humid climate area with subtropical to alpine ice sources. The area experiences abundant rainfall in summer and is warm in winter. The vertical differentiation of the mountain forest ecology of rural areas is obvious, and the mountain is divided into four sections from low to high: temperate trees, cold-resistant shrubs, bare rock, and frozen peaks, with snow throughout the year; it has rich plant resources and high biological diversity due to its three-dimensional climate and unique landform [13, 25].

Tibet is a nation with a long history, mainly located on the Qinghai-Tibet Plateau, and a rich knowledge of traditional culture. Plants occupy an important position in Tibetan culture and are used in medicine, food, architecture, textiles, and religious rituals [19, 25, 27]. Tibetans in the Rongjia River Valley have rich and unique knowledge of the local environment. All residents of the township are Tibetan, and the local Tibetan language is the Wei-Tibetan dialect [25]. The income of the Tibetan people in the Rongjia Township mainly comes from grazing, labor exports, border trade, mining of cordyceps, and national subsidies for border residents [25]. Before construction of the road in 2015, travel conditions in and out of the Rongjia River Valley were very poor, and traffic was inconvenient. Currently, there is no vegetable market in Rongjia River Valley, and residents live in the villages of Cang-mu-jian, Da-cang, and Chenthang (Fig. 1). Cang-mu-jian village is located in the middle of the Rongjia Township against a huge mountain, which has an altitude of about 3400 m, an alpine canyon landform, and the main river in the territory from the northeast to the southwest. Da-cang village is located southwest of Dingri County at an altitude of approximately 4060 m. It is an alpine ecosystem that is rich in plant resources. At an altitude of 3667 m, Chenthang Village is distributed with a rich ecosystem, and it is a key area for biodiversity conservation.

### Ethnobotanical surveys

Ethnobotanical field surveys were conducted between August 2021 and June 2023. This research was conducted after obtaining permission from the local government and community committee. During the survey, we explained our purpose to community leaders and requested their assistance, which included providing local guides, translators, and other necessary aid. We interviewed 161 participants (93 men and 68 women) using the snowball method. Prior to each interview, verbal informed consent was obtained, and the International Society of Ethnobiology (ISE) Code of Ethics (2006) was followed (https://www.ethnobiology.net). Ethnobotanical field surveys were conducted using semi-structured interviews and participatory observations. Semi-structured interviews were conducted with the local people, and the questions asked were as follows:

1. Would you mind listing the wild plants that you often consume?
2. Which parts were used, such as the root, stem, leaf, and others?
3. Why do you use this species?
4. How do you cook these plants?
5. Where and when did you collect it?
6. How are the plants used beyond food? Can you share any traditional stories or mythologies associated with them?

We documented the ethnobotanical information for each plant, including the scientific name, vernacular name, parts used, habitat, consumption method, and other uses and tabulated the documented information for these plants (Table 2). We collected 1–3 voucher specimens for each species, and the specimens were identified and preserved in the herbarium of the Kunming Institute of Botany, Chinese Academy of Sciences (KUN). The attributed scientific names were checked using “plants of the world online” (https://powo.science.kew.org/).

### Statistical analysis

Ethnobotanical quantitative indices of the use report (UR) and relative frequency of citations (RFC) were adopted in this study.

Use report (UR) is the specific use of a species cited by an informant. The number of UR can reflect the number of mentions of a species by locals.

The RFC was used to quantify the frequency of use of certain species and was determined using the following formula:

$${\text{RFC = }}\frac{{{\text{FC}}}}{{\text{N}}}$$

FC refers to the number of informants who mentioned a particular wild edible plant, and N represents the number of informants participating in the survey. RFC values vary from 0 to 1, and the higher the RFC value, the more important and valuable the plant is in the area. The RFC value was used to indicate the importance of each wild edible plant, and all surveyed WEPs were ranked in order of significance [26–28].

## Results

### Characteristics of informants

This study interviewed 161 informants from three communities in the Rongjia Township (Table 1). Among the informants, 106 were men and 55 were women. The number of male informants (65.84%) was almost twice as high as that of female informants (34.16%). When we used the snowball sampling method to select informants, they mostly recommended men. In addition, in the process of our household interviews, men in the family usually came forward to be interviewed, whereas women often did not. The ages of the informants ranged from 18 to 99 years. The mean age of the informants was 51 years. All informants of the township are Tibetan.

**Table 1:** Demographic details of the informants

Characteristics	Number	Percentage%
*Communities*		
Chenthang Village	63	39
Cang-mu-jian Village	50	31
Da-cang Village	48	30
*Gender*		
Male	106	66
Female	55	34
*Age*		
18–30	9	6
31–40	21	13
41–50	29	18
51–60	48	30
61–70	30	18
71–80	12	7
81–90	8	5
> 90	4	3
*Ethnicity*		
Tibetan	161	100

### Diversity of WEPs in the Rongjia River Valley

**Fig. 2:**
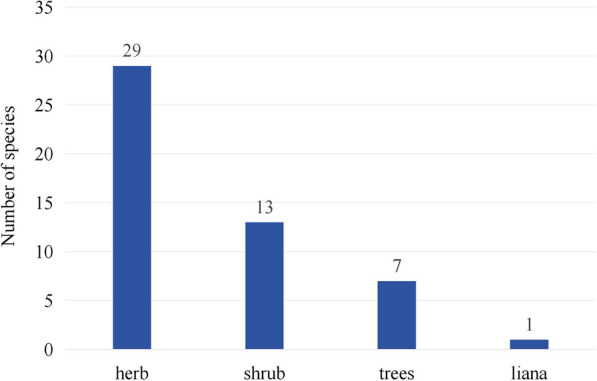
Life forms of WEPs

Fifty wild edible plant species belonging to 28 families and 42 genera were identified (Table 2). The results showed that the most frequently mentioned family was *Rosaceae* (seven species), followed by *Amaryllidaceae* (four species), *Brassicaceae*, *Ericaceae*, *Grossulariaceae*, and *Polygonaceae*, each containing three species. Five families comprised two species. The remaining 17 families contained only one species each (Table 2). At the genus level, the most common genus was *Allium* (four species), followed by *Ribes* (three species), *Lindera* (two species), *Rosa* (two species), and *Zanthoxylum* (two species). The life forms of these WEPs were mostly herbs (29 species) and shrubs (13 species) (Fig. 2).

**Table 2:** Wild edible plants in Rongjia River Valley

Family	Scientific name	Chinese name	Vernacular name	use part	Life form	collect months	Food categories	Local use	Additional local use(s)	UR	RFC	Voucher number
Amaranthaceae	*Chenopodium album* var. *viride* (L.) Pursh	藜	ni-nei	Leaves	Herb	4–5	Vegetable	Made into soup or stir-fried		83	0.52	EBT-RX-50
Amaranthaceae	*Dysphania schraderiana* (Schult.) Mosyakin & Clemants	菊叶香藜	zheng-jiong-chei-du-ba	Leaves	Herb	6–7	Vegetable	Stir-fried		15	0.09	EBT-RX-67
Amaryllidaceae	*Allium wallichii* Kunth	多星韭	guo-ba-rang-dang-ma	Aerial part	Herb	4–5	Seasoning	Add chili peppers and grind them into a seasoning		86	0.53	EBT-RX-1
Amaryllidaceae	*Allium przewalskianum* var. *planifolium* Regel	青甘韭	guo-ba-rang-ba	Aerial part	Herb	4–5	Seasoning	Add chili peppers and grind them into a seasoning		52	0.32	EBT-RX-55
Amaryllidaceae	*Allium paepalanthoides* Airy Shaw	天蒜	sei-bu-lei	Roots	Herb	7	Seasoning	Add chili peppers and grind them into a seasoning		30	0.19	EBT-RX-108
Amaryllidaceae	*Allium fasciculatum* Rendle	粗根韭	ru-guo	Aerial part	Herb	4–5	Seasoning	Add chili peppers and grind them into a seasoning		21	0.13	EBT-RX-77
Apiaceae	*Heracleum nyalamense* R.H.Shan & T.S.Wang	聂拉木独活	kong-dang	Stems	Herb	6—8	Vegetable	Eat it directly after peeling		90	0.56	EBT-RX-3
Apiaceae	*Eriocycla nuda* var. *purpurascens* R.H.Shan & C.C.Yuan	紫花裸茎绒果芹	mang-gei	Leaves	Herb	6–7	Vegetable	Make steamed stuffed bun stuffing		64	0.4	EBT-RX-27
Apocynaceae	*Cynanchum auriculatum* Royle ex Wight	牛皮消	wo-ma-jiong-jiong	Fruit	Shrub	9	Fruit	Eaten freshly		27	0.17	EBT-RX-74
Araceae	*Arisaema erubescens Schott	一把伞南星	tuo-jiong	Stems	Herb	6–9	Substitute grain	Eat it directly after peeling		29	0.18	EBT-RX-28
Araceae	*Pinellia ternata (Thunb.) Makino	半夏	tuo-jie	Seeds	Herb	7–8	Substitute grain	Making cakes		27	0.17	EBT-RX-32
Brassicaceae	*Capsella bursa-pastoris* Medik	荠	ou-jia	Roots and leaves	Herb	6–7	Vegetable	Boiled		66	0.41	EBT-RX-78
Brassicaceae	*Eutrema scapiflorum* (Hook. f. & Thomson) Al-Shehbaz, G. Q. Hao & J. Quan Liu	单花荠	qia-ma-la-mu	Lleaves	Herb	6–8	Vegetable	Stir-fried		17	0.11	EBT-RX-113
Brassicaceae	*Cardamine lyrata* Bunge	水田碎米荠	cha-bu-jiu	Stems	Herb	4–6	Vegetable	Stir-fried		10	0.06	EBT-RX-110
Caprifoliaceae	*Nardostachys jatamansi* (D. Don) DC	甘松	bang-bu	Stem and leaves	Herb	6–7	Seasoning	Add chili peppers and grind them into a seasoning	Cultural: Ritual(leaves)	80	0.50	EBT-RX-100
Caryophyllaceae	*Stellaria aquatica* Scop	鹅肠菜	jia-ba-la-mu	Leaves	Herb	4–5	Vegetable	Made into soup or make steamed stuffed bun stuffing		30	0.19	EBT-RX-107
Coriariaceae	*Coriaria terminalis* Hemsl	草马桑	guo-ma-en-jiong	Fruit	Herb	9	Fruit	Eaten freshly		59	0.37	EBT-RX-102
Cucurbitaceae	*Momordica cochinchinensis* Spreng	木鳖子	gu-jiu-ru-bai	Aerial part	Liana	7–8	Vegetable	Made into soup or stir-fried		29	0.18	EBT-RX-109
Dennstaedtiaceae	*Pteris* Gled. ex Scop	凤尾蕨属	tong-xia	Leaves	Herb	5–8	Vegetable	Stir-fried		38	0.24	EBT-RX-92
Dryopteridaceae	*Dryopteris barbigera* (T. Moore et Hook.) O. Ktze	多鳞鳞毛蕨	che-jiu-wa	Leaves	Herb	6–7	Vegetable	Stir-fried		14	0.09	EBT-RX-4
Ericaceae	*Rhododendron anthopogon* D. Don	髯花杜鹃	po-lu	Stem and leaves	Shrub	6–7	Vegetable	Eat it directly	Cultural: Ritual(leaves); Medicine: Cold(leaves), Begma(leaves)	51	0.32	EBT-RX-17
Ericaceae	*Cassiope fastigiat*a (Wall.) D. Don	扫帚岩须	ba-jia-ba	Aerial part	Shrub	5–6	Vegetable	Boiled		31	0.19	EBT-RX-83
Ericaceae	*Vaccinium fragile* Franch	乌鸦果	na-mu-di-di	Fruit	Shrub	9	Fruit	Eaten freshly		28	0.17	EBT-RX-69
Fabaceae	*Caragana bicolor* auct.non Kom	二色锦鸡儿	cha-ma	Leaves	Shrub	5–8	Vegetable	Boiled		27	0.17	EBT-RX-8
Gentianaceae	*Swertia bifolia* Batalin	二叶獐牙菜	gei-duo-ba	Whole plants	Herb	8–9	Vegetable; Healthcare food	Boiled	Medicine: Cold(leaves), Begma(leaves)	14	0.09	EBT-RX-115
Grossulariaceae	*Ribes alpestre* Wall. ex Decne	长刺茶藨子	giu-lu	Fruit	Shrub	8–9	Fruit	Eaten freshly	Cultural: Dyes(fruits)	102	0.63	EBT-RX-48
Grossulariaceae	*Ribes orientale* Desfontaines	东方茶藨子	a-kang-bu	Fruit	Shrub	9	Fruit	Eaten freshly		97	0.6	EBT-RX-20
Grossulariaceae	*Ribes takare* var. *desmocarpum* (Hook.f. & Thomson) L.T.Lu	束果茶藨子	yi-bi-rang-bu	Fruit	Trees	8–9	Fruit	Eaten freshly		16	0.10	EBT-RX-60
Lamiaceae	*Thymus quinquecostatus* Čelak	地椒	ga-ruo-ma-zi	Fruit	Shrub	7–8	Seasoning	Add chili peppers and grind them into a seasoning		33	0.21	EBT-RX-25
Lauraceae	*Lindera nacusua* (D. Don) Merr	绒毛山胡椒	lu-guo	Fruit	Trees	9	Seasoning	Add chili peppers and grind them into a seasoning		85	0.53	EBT-RX-80
Lauraceae	*Lindera kariensis* W. W. Smith	更里山胡椒	rang-dang-ma	Fruit	Shrub	9	Seasoning	Add chili peppers and grind them into a seasoning		62	0.39	EBT-RX-81
Malvaceae	*Malva pusilla* Sm	圆叶锦葵	jiang-ba-la-mu	Leaves	Herb	7–8	Vegetable	Stir-fried		55	0.34	EBT-RX-51
Ophioglossaceae	*Sceptridium daucifolium* (Wall. ex Hook. & Grev.) Lyon	薄叶阴地蕨	pie-la-ba	Leaves	Herb	5–8	Vegetable	Boiled		38	0.24	EBT-RX-22
Orchidaceae	*Satyrium nepalense var. *ciliatum* (Lindl.) Hook. f	缘毛鸟足兰	tu	Stems	Herb	8–9	Substitute grain	Eat it directly after peeling		28	0.17	EBT-RX-26
Polygonaceae	*Koenigia polystachya subsp. Wall.* ex Meisn.) T.M.Schust. & Reveal	多穗蓼	long-ma	Leaves	Shrub	6–7	Vegetable	Boiled	Animal Food: Fodder(aerial part)	49	0.30	EBT-RX-104
Polygonaceae	*Rheum nobile* Hook.f. & Thomson	塔黄	cha-ba	Stems	Herb	7–8	Vegetable	Eat it directly after peeling		8	0.05	EBT-RX-105
Polygonaceae	*Bistorta macrophylla* (D.Don) Soják	圆穗蓼	ren-bu	Leaves	Herb	6–7	Vegetable	Stir-fried		4	0.02	EBT-RX-16
Pteridaceae	*Coniogramme japonica* (Thunb.) Diels	凤了蕨	a-wu-di-wu	Leaves	Herb	6–7	Vegetable	Boiled		4	0.02	EBT-RX-118
Ranunculaceae	*Thalictrum foetidum* L	腺毛唐松草	gu-bu	Aerial part	Herb	6–7	Vegetable	Made into soup or stir-fried		71	0.44	EBT-RX-62
Rhamnaceae	*Berchemia edgeworthii* Lawson	腋花勾儿茶	pie-shi-zi	Leaves	Shrub	5–6	Beverage	Soak water to drink		28	0.17	EBT-RX-73
Rosaceae	*Rosa sericea* var. *glandulosa* Osmaston	腺叶绢毛蔷薇	sei-ri-ma	Fruit	Trees	7	Fruit	Eaten freshly	Fuel: Firewood(roots)	122	0.76	EBT-RX-24
Rosaceae	*Rosa macrophylla* var. *glandulifera* T.T.Yu & T.C.Ku	腺果大叶蔷薇	sei-bang-duo	Fruit、roots	Shrub	7	Fruit	Eat it directly after peeling	Construction: Thatch(roots)	110	0.68	EBT-RX-43
Rosaceae	*Fragaria nubicola* Lindl. ex Lacaita	西藏草莓	ding-ba-jia-luo	Fruit	Herb	9	Fruit	Eaten freshly		109	0.68	EBT-RX-9
Rosaceae	*Prunus mira* Koehne	光核桃	kang-bu	Fruit	Trees	8–9	Fruit	Eaten freshly		105	0.65	EBT-RX-89
Rosaceae	*Rubus* L	悬钩子属	nia-niu-ma	Fruit	Shrub	9	Fruit	Eaten freshly		27	0.17	EBT-RX-85
Rosaceae	*Potentilla* L	委陵菜属	rang-ba	Leaves	Herb	6–7	Vegetable	Boiled		26	0.16	EBT-RX-31
Rosaceae	*Sorbus vestita* (Wall. ex G. Don) S. Schauer	白叶花楸	ge-ru-bai	Fruit	Trees	8–9	Fruit	Eaten freshly		16	0.10	EBT-RX-21
Rutaceae	*Zanthoxylum bungeanum* Maxim	花椒	ei-ma	Fruit	Trees	9	Seasoning; Healthcare food	Add chili peppers and grind them into a seasoning	Medicine: Rheumatism(fruits), Sore throat(fruits)	121	0.75	EBT-RX-46
Rutaceae	*Zanthoxylum acanthopodium* DC	刺花椒	cha-dong	Fruit	Trees	6–7	Seasoning; Healthcare food	Add chili peppers and grind them into a seasoning	Medicine: Toothache(fruits), Rhinitis(fruits)	106	0.66	EBT-RX-90
Urticaceae	*Urtica hyperborea* Jacquem. ex Wedd	高原荨麻	sa-du-ba	Fruit	Herb	4–5	Vegetable	Consumed as a soup		115	0.71	EBT-RX-54

**Fig. 3:**
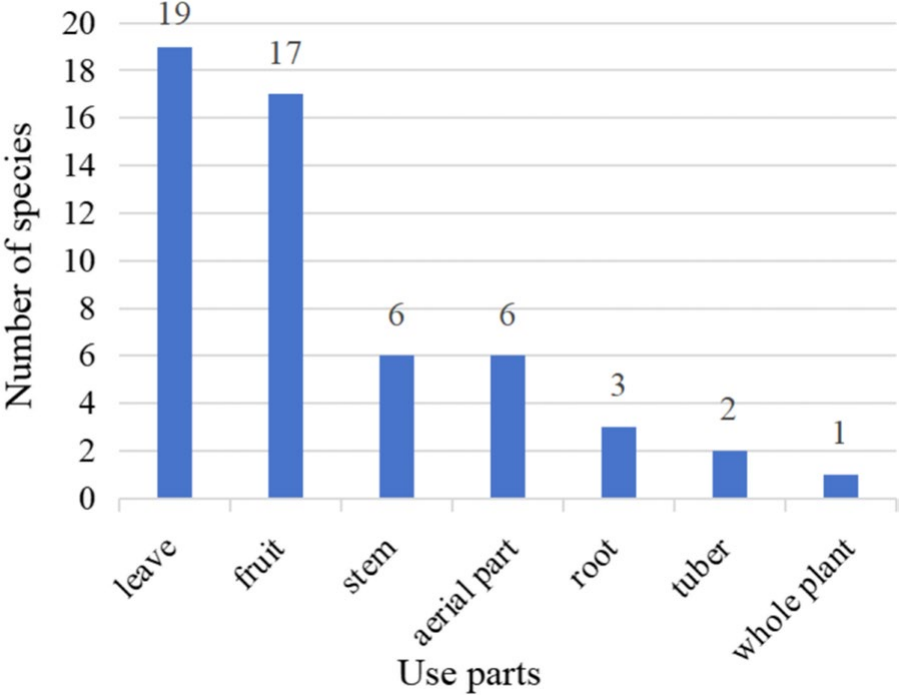
Edible parts of WEPs

We found that many parts of the plants were consumed, such as the leaves, fruits, stems, seeds, and roots (Fig. 3). The most commonly consumed part was the leaves (19 species), followed by fruits (17 species) and stems (six species). Wild vegetables and fruits were the two main categories of WEPs. The parts used for wild vegetables were the leaves, whereas the parts used for wild fruits were the fruits.

Based on the information provided by the participants, we summarized the WEPs into six categories (Table 3). The WEPs consumed by the Tibetan people in the Rongjia River Valley included vegetables, fruits, spices, healthcare food, substitute grains, and beverages. Wild vegetables were the most commonly consumed (24 species), followed by wild fruits (12 species).

**Table 3:** Use reports and use categories

Criteria	Use categories	Number of species	Use report (UR)
Plants material what were used to cook dishes (including making salads directly with raw plant material)	Vegetable	24	1009
Fruits that were only eaten when they were ripe, similar to apple, pear and strawberry	Fruit	12	831
Plants that could be added to dishes or soups to increase the flavor of food	Seasoning	10	317
Not only edible plants, but could also be used by local people to treat diseases	Healthcare food	3	241
Plants that could be used as a direct starch supplement or processed into starch	Substitute grain	3	89
Plants that could be processed into home-made liqueurs or alcoholic beverages and processed into herbal teas	Beverage	1	12

**Fig. 4:**
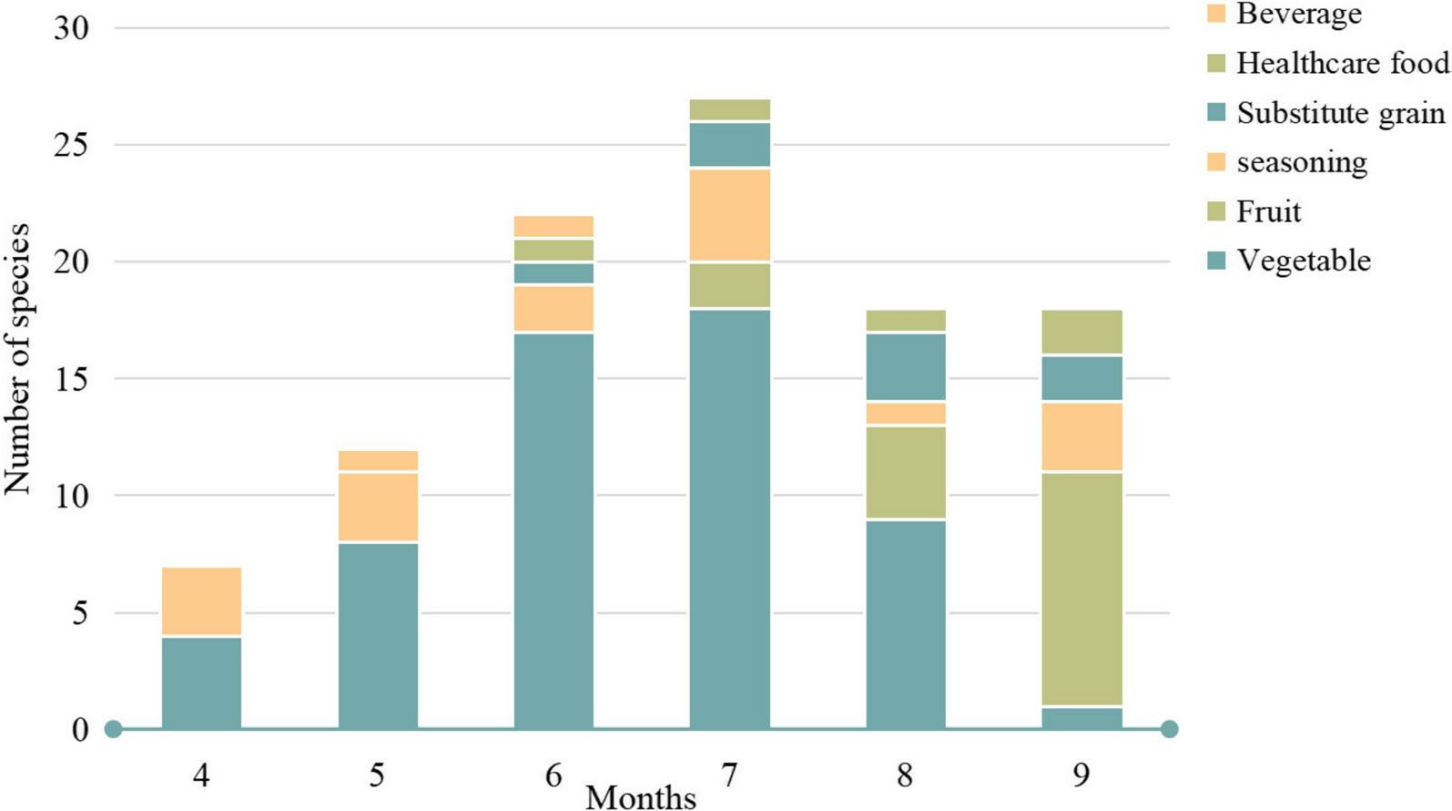
Months of collection for WEPs

The WEPs were typically collected from April to September (Fig. 4). The collection time for WEPs depended on the maturation of the parts used. Most of the wild vegetables were collected from June to July. Wild fruit was collected primarily from July to September.

### Vegetables

Half of all species were wild vegetables (24 species) belonging to 17 families: *Brassicaceae* and *Polygonaceae* (each having three species); *Amaranthaceae*, *Apiaceae*, and *Ericaceae* (each having two species); and the remaining 12 families each having one species. The main edible parts were the tender leaves and stems. Wild vegetables were usually collected from April to September and used only for the family’s own consumption, not for sale. The processing method was found to be relatively simple, usually involving boiling and then stir-frying or making soups. The three most frequently mentioned species were *U. hyperborea* (RFC = 0.71), *Heracleum nyalamense* R. H. Shan & T. S. Wang (RFC = 0.56), and *Chenopodium album* var. *viride* (L.) Pursh (RFC = 0.52).

*U. hyperborea* is a seasonal vegetable, and Tibetans in the Rongjia River Valley only collect and consume it from April to May. Because *U. hyperborea* stems and leaves have sharp thorny hairs, they have unique collection and processing methods. Most locals wear gloves and use scissors to collect the tender leaves of *U. hyperborea* for consumption. To handle the sharp thorny hair, they were rinsed slightly with boiling water prior to cooking. After boiling, the prickly hair of *U. hyperborea* did not cause any irritation to the skin; therefore, *U. hyperborea* was added to the broth and cooked into a thick soup. During the interviews, many informants (*n* = 64) said that they had to eat *U. hyperborea* once a year.

*H. nyalamense* is endemic to Tibet, and Tibetans in the Rongjia River Valley collect it annually from June to August. The stem can be peeled and then eaten raw. Interestingly, when local people go up to the mountains, they consume *H. nyalamense* as a snack.

*C. album* var. *viride* can be consumed in several ways. After collection from April to May, its tender leaves can be cooked, fried, steamed, stuffed into buns, or added into a broth like *U. hyperborea* to make a thick soup.

### Fruits

Fruits (12 species) were the second largest use category of WEPs, belonging to five families: *Rosaceae* (six species), *Grossulariaceae* (three species), and the remaining three families each having one species. Wild fruits were found to be available from June to September, and all fruits were used only for the family’s consumption and not for sale. They were typically consumed as fresh fruits, such as snacks, and their main edible parts were fruits. The three most frequently mentioned species were *Rosa sericea* var. *glandulosa* Osmaston (RFC = 0.76), *Rosa macrophylla* var. *glandulifera* T. T. Yu & T. C. Ku (RFC = 0.68), and *Fragaria nubicola* Lindl. ex Lacaita (RFC = 0.68), which was observed throughout the Rongjia River Valley.

*R. sericea* var. *glandulosa* and *R. macrophylla* var. *glandulifera* are wild fruits of the Rosaceae family that ripen in July. These two wild fruits are very similar in appearance. *R. macrophylla* var. *glandulifera* requires peeling and removal of the small thorns before consumption, while *R. sericea* var. *glandulosa* can be consumed directly.

*F. nubicola* matures in September and typically thrives in habitats such as ditches, forest areas, and hillside grasses. The ripe fruit can be eaten directly as wild fruit. Thus, these three plants are important and readily available as wild fruits for vitamin C supplementation between July and September.

### Seasoning

Seasoning are an important food category for WEPs in the Rongjia River Valley, with ten documented species belonging to five families: *Amaryllidaceae* (four species), *Lauraceae* and *Rutaceae* (having two species each), and the remaining two families each having one species. Data were collected from April to September. The main edible parts of these plants were found to be the fruits. The three most frequently mentioned species were *Zanthoxylum bungeanum* Maxim. (RFC = 0.75), *Zanthoxylum acanthopodium* DC. (RFC = 0.66), and *Allium wallichii* Kunth (RFC = 0.53).

**Fig. 5:**
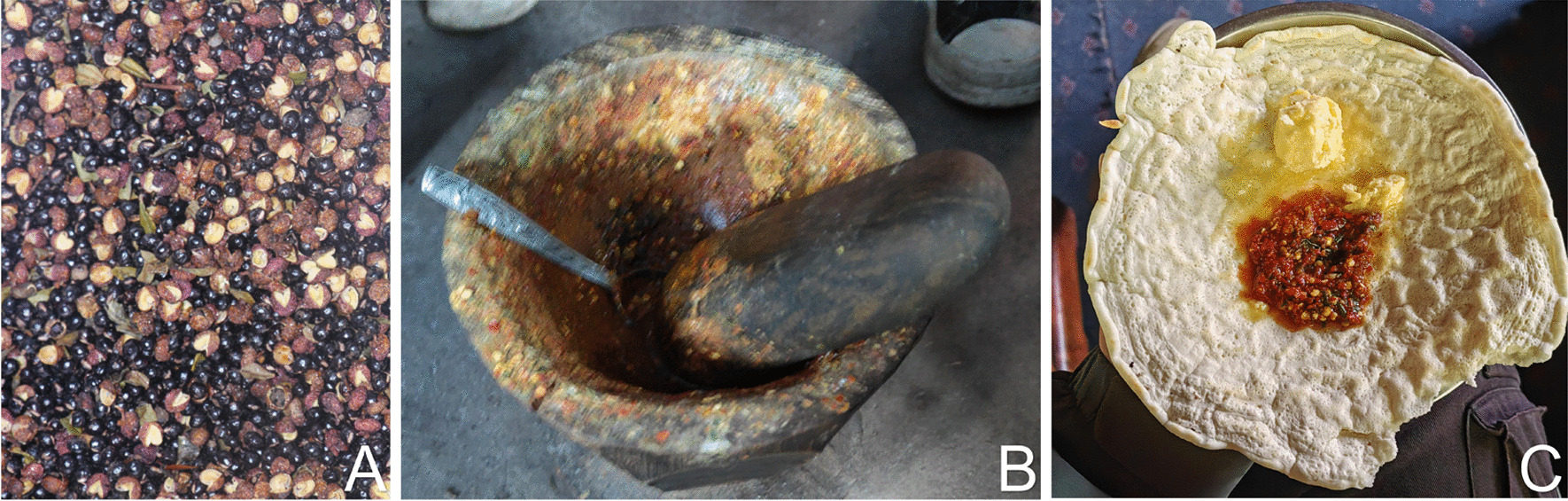
**A** The most commonly used seasoning, *Zanthoxylum bungeanum* Maxim. **B** Tools used for grinding wild seasoning plant. **C** A homemade seasoning used for serving with flatbread

The ten seasoning plants were used in a similar manner by the local people, grind seasoning plants, adding chili peppers mixed for seasoning (Fig. 5). Potatoes are the main food crop in the Rongjia River Valley, and it is common practice to season them with these homemade spice blends. In addition to potatoes, locals use these homemade spice blends for their various other staples. Although the method of consumption is consistent, distinct plants and proportions produce diverse and delicious flavors cherished by the local people. Notably, *A. wallichii*, which has an excellent flavor, has been introduced into home gardens by the local people.

### Substitute grains

Three substitute grains were also observed, including *Arisaema erubescens* Schott (RFC = 0.18), *Pinellia ternata* (Thunb.) Makino (RFC = 0.17), and *Satyrium nepalense* var. *ciliatum* (Lindl.) Hook. f*.* (RFC = 0.17).

From June to September, *A. erubescens* tubers are collected by the local people and are subsequently peeled and served. One informant reported that "after eating *A. erubescens* once, we have completed a great event in our life." We also found that before the liberation of the Rongjia Township, some farmers would go to their landlord's house to help them hoe the fields for free in order to collect *A. erubescens* from the land. At that time, the local people were very dependent on *A. erubescens*.

The consumption of *P. ternata* is special. Its seeds are collected from July to August and are washed, ground, wrapped, and buried for seven days. Finally, the mixture is stirred with highland barley flour to prepare dough bread.

Although reports were collected (*n* = 11), we discovered that *A. erubescens* and *P. ternata* are no longer utilized. However, in the former Rongjia River Valley, these were important substitutes for grain plants.

*S. nepalense* var. *ciliatum* is consumed in a unique manner. Locals collect their tubers from August to September every year, wash and steam them, remove the skin, and grind them into a cylindrical shape—similar to that of sausage—before consumption.

### Other categories: healthcare food and beverages

Five WEPs from other food categories, including healthcare food and beverages, were identified. Four species were found to have medicinal value and have been used to treat rheumatism, sore throat, toothache, rhinitis, cold, and begma. The three most frequently mentioned healthcare foods were *Z. bungeanum* (RFC = 0.75), *Z. acanthopodium* (RFC = 0.66), and *Rhododendron anthopogon subsp. anthopogon* (RFC = 0.32).

*Z. bungeanum* and *Z. acanthopodium* are simultaneously used as seasoning and healthcare food by Tibetans in the Rongjia River Valley. After chewing *Z. bungeanum*, it is applied for toothache, having an analgesic effect, while soaking it in water is said to prevent rheumatism. Some informants (*n* = 6) mentioned that for people with hypertension, it is necessary to use *Z. bungeanum* as little as possible, as it can lead to increased blood pressure.

*R. anthopogon subsp. anthopogon* leaves are collected in June and July of each year and are chewed as a snack by Tibetans in the Rongjia River Valley. Soaking the leaves of *R. anthopogon subsp. anthopogon* in water is employed to treat colds and begma. These medicinal plants are important for residents to improve their health and prevent diseases.

Only one wild plant was used as a beverage by the Tibetans in the Rongjia River Valley: *Berchemia edgeworthii* Lawson. Local people collect *B. edgeworthii* leaves in May and June of each year, which are then dried, stored, and soaked in drinking water.

## Discussion

### Plants used in the past mirror fading life memories

Very little documentation could be retrieved about the Tibetan people living in the Rongjia Township, with limited local county records [29]. Plants, as vessels of human understanding of the natural world, bear the imprints of people’s past experiences and practices. From the interaction between plants and humans, we can see part of the microcosm of historical development and provide a reasonable explanation for the motivating force of social evolution and its mechanism of action [30].

During this investigation, we asked the participants to list the WEPs they used. The older informants (*n* = 6) actively told us much about the history of WEPs when referring to *A. erubescens*. Before the Rongjia Township was liberated, local people would voluntarily apply to plow the land for the landlords, and they would not ask for payment, as they wanted to collect *A. erubescens* for consumption. Interestingly, *A. erubescens* has been developed and used as a drug[31] with a bitter and astringent taste, and studies have shown that *A. erubescens* tuber starch content is 28%, but it is toxic and inedible[32–34]. However, in their opinion, *A. erubescens* is the most delicious food, and it is a great occasion to be able to eat *A. erubescens* once in their lifetimes.

In the limited literature, we understand that local people were once part of a serfdom system. To survive, oppressed locals made efforts to discover certain natural plants that could serve as important substitutes for grains. To ensure an adequate food supply, it was deemed necessary to eat as many plant species as possible [30]. From the descriptions of *A. erubescens* as a delicious food source, we can see how difficult it was for people to survive in the past and how important WEPs were to them. The use of these plants has formed a social memory in that generation.

Therefore, although *A. erubescens*, *P. ternata*, and *S. nepalense* var. *ciliatum* are no longer collected and used, we believe that the related accounts are meaningful because this traditional knowledge can be seen as a kind of social memory that reflects the close relationship between people and plants. We also suggest that for plants that were used in the past but are no longer used or even barely remembered, we need to consider the social context of the past to understand the story in depth, because the study of the interactions between humans and plants is one of the subject objectives of ethnobotany.

### Services provided by WEPs collected by locals

WEPs are an important part of the daily diet in many remote and underdeveloped areas [35, 36]. The demand for WEPs is closely related to the shortage of cultivated food resources. According to information provided by the township head, the annual output of grain per capita in the Rongjia Township is 189.04 kg. This is far below the standard set by the World Food Program (WFP), which states that about 400 kg of grain per capita per year is necessary to maintain a healthy living and work environment. Under normal circumstances, when the food supply is destroyed by events such as famine, extreme weather, and earthquakes, WEPs play an important role in supplementing staple foods [37]. Tubers of *A. erubescens* have been used as a food substitute to supplement carbohydrates in times of poverty and food shortages as emergency rations by the Tibetans in the Rongjia River Valley. Some studies have shown that in the dried tubers of *A. erubescens*, the starch content is 52.91% and the amylopectin content is 29.1–32.0% [38]. These substitute grains are crucial for Tibetans in the Rongjia River Valley in dealing with food shortages. Thus, the collection and utilization of WEPs by the local people can enhance their resistance to local food systems [21].

In remote areas where transportation is difficult, a variety of wild fruits and vegetables are used as vitamins, minerals, and dietary fiber supplements [39]. Due to the snowy mountain barrier, it is difficult for residents in the Rongjia Township to reach the county market. Vitamins, minerals, plant proteins, and other nutrients must be obtained from the natural environment. From April to September, many WEPs are collected and eaten by Tibetans in the Rongjia River Valley. Several studies have shown that WEPs provide nutrients to humans and have revealed the nutrients contained in these plants. For example, the most commonly used plant, *U. hyperborea*, has a high nutritional value. Studies have shown that its crude protein content is up to 36.4% [40, 41], and it is rich in essential amino acids such as lysine and tryptophan, which cannot be synthesized and are indispensable for humans and animals. Moreover, it contains large amounts of minerals, such as Ca, Mg, K, Fe, Zn [41], carotene, and vitamin B [42].

In addition to wild vegetables, wild fruits provide local people with vitamins, minerals, and other nutrients. Due to the limited cultivated land area, there is only one cultivated fruit tree, *Prunus mira Koehne*, which is very similar to several other Himalayan River Valley regions [4–6, 8, 21], such as the Yadong River Valley [4] and Gyirong River Valley [6, 8]. Local people in these areas can rely only on wild fruit to meet their nutritional needs. For example, the Rongjia River Valley is rich in wild fruit resources from the genus *Ribes*. The nutrient content of wild fruits of this genus is characterized by low fat and high vitamin C, vitamin E, and potassium contents [43, 44]. For example, the ripe fruit of *R. sericea var. glandulosa* and *R. macrophylla* var. *glandulifera* is rich in vitamins and dietary fiber and contains 16 types of amino acids, including seven essential amino acids. The total amount of amino acids is more than 3500 mg per 100 g, and the fruit is rich in mineral elements, especially Ca, K, Fe, and Zn [45]. The fruit also contains large amounts of vitamin C and has a moderate sugar-to-acid ratio, and the content of essential amino acids and trace elements can meet the nutritional requirements of the human body. Thus, it is a high-quality wild fruit in terms of taste and nutrition [46]. At the same time, some wild vegetables also contain unique physiologically active substances and have extremely high medicinal value and corresponding healthcare functions [48–50]. A previous pharmacological study showed that extracts of *U. hyperborea* significantly reduced uric acid levels [50], which is notable because hyperuricemia and gout are widespread afflictions. More importantly, *U. hyperborea* contains various bioactive substances, such as polyphenols, flavonoids, and polysaccharides [51]. Thus, it has anti-inflammatory and antibacterial properties and improves immunity and other important physiological functions. This plant has the important effects of maintaining health, reducing uric acid, and supplementing nutrition. It is commonly found in high-altitude plateau regions and holds the potential for further development.

Some WEPs not only make a positive contribution to dietary structure and nutritional supplementation but also serve as medicines for healthcare [35, 52]. The leaves of *R. anthopogon* and *S. bifolia* can be used to treat colds and coughs. The fruits of *Z. bungeanum* have been used to treat rheumatism and sore throat, while the fruits of *Z. acanthopodium* can be used to treat toothache and rhinitis. Recent studies demonstrated that aqueous extracts of *Z. bungeanum* may play a therapeutic role in rheumatoid arthritis [53, 54].

WEPs also have cultural value. Vegetables have certain social and cultural carrier functions and play a role in promoting communication within the community [55]. The Rongjia River Valley was the place of death for Master Milarepa, founder of the Kagyu School of Tibetan Buddhism [56]. Master Milarepa ate wild *U. hyperborea* daily during his penance period. Currently, the Tibetan people of the Rongjia River Valley eat *U. hyperborea* once a year to commemorate Master Milarepa. People from different regions have strong regional and cultural characteristics that influence their choice and use of WEPs [5]. Wild plants provide food for the community, and the tradition of using WEPs has become part of the culture of these communities [57].

Compared to other studies on WEPs, fewer plant species were documented in this study [5, 7, 18]; however, these plants have become integral to the way of life and survival strategies of the community, indicating a deep historical and cultural connection. Tibetans living in the Rongjia River Valley, one of the most remote and underdeveloped areas in the world, have received many benefits from WEPs to support their daily lives.

### WEPs could still provide benefits in future

With China’s rapid economic development, the government is paying increasing attention to the development of the border and remote areas. In the context of poverty alleviation in China, the living conditions of residents have greatly improved. In 2015, construction of the road was completed and opened to traffic, enabling the transport of external materials to the valley. The goods transported into the Rongjia Township slowly appeared on the dining tables of the local Tibetans, and the arrival of rice and white flour halted the collection and utilization of *A. erubescens*, *P. ternate*, and *S. nepalense* var. *ciliatum.*

With the help of the government and the efforts of the locals, the growing tourism industry has raised the income and living standards of the residents of the Rongjia River Valley. Despite improvements in living conditions, the Tibetan people in the Rongjia River Valley still have to encounter and overcome many unstable factors. For example, the area was hit by a 5.9-magnitude earthquake in 2015. Even in May 2023, we encountered heavy snow blocking the mountain when entering the area for our research, and access to and from the Rongjia River Valley remains hampered by extreme weather. Thus, there is still instability in relying solely on the external market supply, and local residents also need to rely on WEPs to supplement their living needs. Wild substitutes for grains, vegetables, fruits, and other natural products can increase the resilience of the local community and enhance survival. Therefore, it is important to document the traditional knowledge of WEPs for communities with minimal global economic exchange, whether in the past, present, or future.

## Conclusion

This study was conducted in the Rongjia River Valley, which is one of the most remote and least developed areas in China and worldwide. We interviewed 161 informants and identified 50 WEP species belonging to 28 families and 42 genera. These plants serve not only as vital sources of grains, vegetables, and fruits for sustaining the daily lives of local residents and enabling them to adapt to challenging environments, but they also hold fading memories of the region’s historical narratives.

Moreover, the utilization of local WEPs enhances the community’s resilience in the face of sudden destabilizing events. Therefore, this study holds great significance in documenting and preserving the knowledge related to traditional WEPs among the Tibetan population in the Rongjia River Valley. Additionally, this study highlights the need to investigate traditional food systems in communities with limited economic interactions with the world, as this can bolster local resilience in times of destabilizing events.

## Data Availability

Please contact the corresponding author for data requests.

## Abbreviations

WEPs: Wild edible plants
UR: Use Report
RFC: Relative frequency of citation
*R. sericea* var. *glandulosa*: *Rosa sericea* Var. *glandulosa* Osmaston
*Z. bungeanum*: *Zanthoxylum bungeanum* Maxim
*U. hyperborea*: *Urtica hyperborea* Jacquem. ex Wedd
*A. erubescens*: *Arisaema erubescens* Schott
*P. ternata*: *Pinellia ternata* (Thunb.) Makino
*S. nepalense* var. *ciliatum*: *Satyrium nepalense* Var. *ciliatum* (Lindl.) Hook.f.
*H**.** nyalamense*: *Heracleum nyalamense* R. H. Shan & T. S. Wang
*C**.** album* var. *viride*: *Chenopodium album* Var. *viride* (L.) Pursh
*R. macrophylla* var. *glandulifera*: *Rosa macrophylla* Var. *glandulifera* T. T. Yu & T. C. Ku
*nubicola*: *Fragaria nubicola* Lindl. ex Lacaita
*Z. acanthopodium*: *Zanthoxylum acanthopodium* DC
*A. wallichii*〹: *Allium wallichii* Kunth
